# Fluorescence ratio imaging of interstitial pH in solid tumours: effect of glucose on spatial and temporal gradients.

**DOI:** 10.1038/bjc.1996.518

**Published:** 1996-10

**Authors:** M. Dellian, G. Helmlinger, F. Yuan, R. K. Jain

**Affiliations:** Edwin L Steele Laboratory, Department of Radiation Oncology, Massachusetts General Hospital, Harvard Medical School, Boston 02114, USA.

## Abstract

**Images:**


British Journal of Cancer (1996) 74, 1206-1215
© 1996 Stockton Press All rights reserved 0007-0920/96 $12.00

### Fluorescence ratio imaging of interstitial pH in solid tumours: effect of glucose on spatial and temporal gradients

M Dellian, G Helmlinger, F Yuan and RK Jain

*Edwin L Steele Laboratory, Department of Radiation Oncology, Massachusetts General Hospital, Harvard Medical School, Boston, Massachusetts 02114 USA.*

**Summary** Tumour pH plays a significant role in cancer treatment. However, because of the limitations of the current measurement techniques, spatially and temporally resolved pH data, obtained non-invasively in solid tumours, are not available. Fluorescence ratio imaging microscopy (FRIM) has been used previously for non-invasive, dynamic evaluation of pH in neoplastic tissue *in vivo* (Martin GR, Jain RK 1994, *Cancer Res.*, **54**, 5670–5674). However, owing to problems associated with quantitative fluorescence in thick biological tissues, these studies were limited to thin (50 $\mu$m) tumours. We, therefore, adapted the FRIM technique for pH determination in thick ($\approx$2 mm) solid tumours *in vivo* using a pinhole illumination–optical sectioning (PIOS) method. Results show that (1) steep interstitial pH gradients (5 $\mu$m resolution), with different spatial patterns, exist between tumour blood vessels; (2) pH decreased by an average of 0.10 pH units over a distance of 40 $\mu$m away from the blood vessel wall, and by 0.33 pH units over a 70 $\mu$m distance; (3) the maximum pH drop, defined as the pH difference between the intervessel midpoint and the vessel wall, was positively correlated with the intervessel distance; (4) 45 min following a systemic glucose injection (6 g kg$^{-1}$ i.v.), interstitial pH gradients were shifted to lower pH values by an average of 0.15 pH units, while the spatial gradient (slope) was maintained, when compared with preglucose values. This pH decrease was not accompanied by significant changes in local blood flow. pH gradients returned to near-baseline values 90 min after glucose injection; (5) interstitial tumour pH before hyperglycaemia and the glucose-induced pH drop strongly depended on the local vessel density; and (6) sodium bicarbonate treatment, either acute (1 M, 0.119 ml h$^{-1}$ for 3 h i.v.) or chronic (1% in drinking water for 8 days), did not significantly change interstitial tumour pH. Modified FRIM may be combined with other optical methods (e.g. phosphorescence quenching) to evaluate non-invasively the spatial and temporal characteristics of extracellular pH, intracellular pH and $pO_2$ in solid tumours. This will offer unique information about tumour metabolism and its modification by treatment modalities used in different cancer therapies.

**Keywords:** interstitium; pH gradient; fluorescence ratio imaging pH is an important determinant of tumour growth, metabolism and response to various treatment modalities such as chemotherapy, radiation therapy, hyperthermia and photodynamic therapy. Therefore, a detailed knowledge about spatial and temporal variations of tumour pH and its modification is needed, especially since marked intra- and intertumour heterogeneities in interstitial pH have been reported (Song *et al.*, 1993). Tumour pH has traditionally been measured with techniques which either (1) are invasive [$H^+$-sensitive electrodes (Ashby, 1966)]; (2) do not provide sufficient spatial resolution [e.g. nuclear magnetic resonance (Daly and Cohen, 1989), positron emission tomography (Hawkins and Phelps, 1988), implantable microchambers (Gullino *et al.*, 1965)]; (3) are not adequate for repeated measurements at a given location (electrodes, microchambers); or (4) do not provide a quantitative analysis of tumour pH with respect to the local haemodynamics.

Recent studies have shown that *in vivo* fluorescence ratio imaging microscopy (FRIM) is a useful technique for pH measurements in living tissues (Kaneko *et al.*, 1991; Martin and Jain, 1993; Mordon *et al.*, 1994). It has been successfully used for the determination of interstitial pH profiles in thin (50 $\mu$m), essentially two-dimensional tumours grown in the rabbit ear chamber (Martin and Jain, 1994). The technique provided adequate spatial and temporal resolutions for the non-invasive determination of pH and its modification owing to treatment such as hyperglycaemia and hypercaepnia (Martin and Jain, 1994). In thick biological tissues, however, the use of quantitative fluorescence microscopy is limited due to: (1) the contribution of out-of-focus light to the tissue slice of interest; (2) significant light scattering and absorption, which not only differ in blood *vs* interstitium, but may also differentially affect the excitation intensities at two different wavelengths used for ratio imaging (Pittman, 1986); and (3) inner filtering and reabsorption phenomena (Tanke *et al.*, 1982; Van Oostveldt and Bauwens, 1990). To circumvent these problems, we developed a pinhole illumination–optical sectioning (PIOS) adaptation of the FRIM technique, to measure interstitial pH in thick, three-dimensional, solid tumours. Using this approach, we measured the following parameters: (1) interstitial pH profiles, at the microscopic level, in human colon adenocarcinoma tumours xenotransplanted in non-anaesthetised, severe combined immunodeficient (SCID) mice; (2) the effect of systemic glucose injection on spatial and temporal pH profiles; and (3) the effect of microcirculation parameters (e.g. intervessel distance and vessel blood flow) on interstitial pH.

### Materials and methods

### *Animal model*

Experiments were performed in severe combined immunodeficient (SCID) mice (6–8 weeks old, 25–30 g), bred and housed in our defined flora animal colony. A dorsal skinfold chamber was surgically implanted under anaesthesia (75 mg ketamine hydrochloride/25 mg xylazine per kg s.c.), as described in detail previously (Leunig *et al.*, 1992). After a 2 day recovery period, a tumour was grown by seeding the dorsal chamber with $\approx 5\times10^5$ human colon adenocarcinoma cells (LS174T; ATCC, Rockville, MD, USA) initially expanded *in vitro*. Tumour growth and microcirculation were monitored through the glass coverslip of the chamber. pH experiments were performed 20–25 days after cell implantation, when tumours had reached a discoid shape of $\approx$5 mm in diameter and 2 mm in thickness.

Correspondence: RK Jain, Department of Radiation Oncology, Cox-7, Massachusetts General Hospital, Boston, MA 02114, USA
Received 11 March 1996; revised 14 May 1996; accepted 16 May 1996

*Fluorescence ratio imaging microscopy (FRIM) in thick tissues*

The imaging station (Figure 1) consisted of an upright microscope (Zeiss Axioplan, Oberkochen, Germany) equipped with transillumination and fluorescence epi-illumination, an intensified CCD camera (C2400-88; Hamamatsu, Bridgewater, NJ, USA), a video monitor (Sony, Montvale, NJ, USA), a video recorder (SVO-9500MD; Sony) and a frame grabber board (Data Translation, Marlboro, MA, USA) for image digitisation on a PC computer (Compaq, Houston, TX, USA). For pH measurements, a solution of free acid 2′,7′-bis-(2-carboxyethyl)-5,6-carboxy-fluorescein (BCECF; Molecular Probes, Eugene, OR, USA) was injected intravenously at a dose of 0.7 mg $kg^{-1}$. The fluorescence emission of BCECF maximally increases with $[H^+]$ at an excitation wavelength of 495 nm and is insensitive to $[H^+]$ changes at 440 nm (isosbestic point). The ratio of the two emission intensities at 535 nm (535$\pm$20 filter, Oriel Co, Stratford, CT, USA) is thus an accurate measure of pH, since it provides intrinsic normalisation for different probe and optical parameters (Bright, 1993). A motorised filter wheel (Oriel Co.) supported the two excitation filters (440$\pm$5 and 495$\pm$5, Oriel Co.).

Quantitative fluorescence microscopy in thick tissue samples is usually hindered by scattering/absorption phenomena and by out-of-focus light contamination (see Introduction). To circumvent these problems, we devised a pinhole illumination–optical sectioning (PIOS) technique. A pinhole diaphragm, in focus with the sample, was introduced in the excitation light path (see Figure 1). With a 20 × long working distance objective (NA 0.40, LD Achroplan, Zeiss), the focused light transmitted through the pinhole resulted in an illumination spot on the sample that was approximately 10 times smaller than the physical size of the pinhole. After investigation of the effect of pinhole size on sampling volume, a 400 μm diameter pinhole (40 μm diameter illumination spot on the sample) was finally chosen (see Results) for subsequent *in vivo* measurements. Fluorescence images within the spot were repeatedly recorded in different tumour locations by moving the specimen with a motorised, $\pm$0.5 μm resolution X–Y stage (Burleigh Instruments, Fishers, NY, USA) controlled by a computer.

*Measurement of microhaemodynamic parameters*

The vessel architecture and blood flow around the areas of interest for local interstitial pH determination were video-taped using transillumination microscopy, the intensified CCD camera, and the VCR, at various times during the experiments. Low light levels and high camera gain were used so that photobleaching of the BCECF probe was negligible. Red blood cell (RBC) velocities and vessel diameters were analysed offline, from the video-tape, using a four-slit photometric apparatus (model 208C; IPM, San Diego, CA, USA) and an image shearing monitor (907; IPM), as described previously (Leunig *et al.*, 1992).

*Experimental protocol*

Following anaesthesia (see above), two tail vein catheters were prepared for each animal; one for the injection of the BCECF probe and the other for glucose injection. The animal was then immobilised in a polycarbonate tube (25 mm internal diameter) on the microscope stage. Using the X–Y stage controller, different tumour locations were recorded and stored for subsequent measurements of pH. A transillumination image of each location was acquired for offline analysis of the local vessel architecture. Background

**Figure 1** Experimental set-up. Excitation pathway: light from the mercury source was filtered (440$\pm$5 or 495$\pm$ nm), directed through the pinhole aperture, and focused on the tissue sample. The specimen was moved using a motorised X–Y stage. Emission pathway: epifluorescence (535$\pm$20 nm) or transillumination images were recorded using an intensified CCD camera, a video monitor and recorder, and a computer.

images (tissue autofluorescence) of all subsequent pH measurement locations were recorded at 440 and 495 nm before dye injection. Fluorescence measurements were initiated once the animal was fully awake for at least 1 h. An initial bolus injection of BCECF (0.7 mg $kg^{-1}$ i.v.) was given. The PIOS scheme was used to record: (1) interstitial pH profiles at preselected locations (acquisition scheme for spatial dynamics); the acquisition consisted of recording two 40 $\mu$m spot images (at 440 and 495 nm) starting from a blood vessel, moving the stage by $20.0 \pm 0.5$ $\mu$m, acquiring the next spot images, until reaching the next blood vessel; the stage was then moved to the next region of interest; and (2) single spot images of the interstitium at various tumour locations, including vascularised, poorly vascularised and non-vascularised areas (acquisition scheme for temporal dynamics). Immediately before or after the fluorescence recordings, transillumination of the area was video-taped (1 min) for offline analysis of the local haemodynamics. The X–Y stage was used to cycle rapidly and repeatedly among the preselected regions. The PIOS scheme was applied again at various time points following glucose injection (0.45 ml, 6 g $kg^{-1}$ i.v.).

### *Sodium bicarbonate study*

In an additional experimental group, animals were treated either chronically for 8 days with 1% sodium bicarbonate added to their drinking water (Gullino *et al.*, 1965), or acutely by i.v. infusion (syringe pump, Harvard Apparatus, South Natick, MA, USA) through the tail vein, for 3 h, of 1 M sodium bicarbonate (0.119 ml $h^{-1}$) dissolved in distilled water. Fluorescence ratios of tumour interstitium were measured in both treated and control (normal drinking water) animals using the PIOS scheme.

### *Image analysis*

Using an image analysis software (NIH Image version 1.58), background images at each wavelength ($I_{back440}$, $I_{back495}$) were subtracted from the corresponding fluorescence images ($I_{440}$, $I_{495}$), and the ratio image, *R*, was computed at each pixel as folows:

$$R = \frac{I_{495} - I_{back495}}{I_{440} - I_{back440}} \quad (1)$$

Profiles shown in Figures 4b and c and 5 were obtained by averaging pixel intensities from $10 \times 5$ $\mu m^2$ rectangular windows within a spot image (spatial resolution of 5 $\mu$m). Ratios were then converted into pH values using an *ex vivo* calibration curve (see below).

### *Calibration of ratio measurements*

Two calibration methods were used; (1) an *in vitro* method, where the fluorescence ratios of BCECF, added to standard pH buffers (mixtures of monobasic and dibasic sodium phosphate at 300 mOsm) in 0.2 mm thick glass capillaries (Vitro Dynamics, Rockaway, NJ, USA), were determined; and (2) an *ex vivo* method, where excised normal subcutaneous tissue and tumour tissue were first incubated for 30 min at 25°C in dye-free buffers of defined pH, then transferred to the same pH buffers mixed with 0.002 mg $ml^{-1}$ BCECF and incubated for 10 min, and finally placed under the microscope for ratio measurements, The optical configuration was the same as the one used for *in vivo* measurements.

### *Statistics*

Results are presented as mean $\pm$ s.e.m. Values of several groups were compared with the Wilcoxon test for dependent groups, and the Kruskal–Wallis and the Mann–Whitney *U*-test for independent groups respectively (Statview; Abacus, Berkeley, CA, USA). The Spearman's correlation coefficient was calculated to analyse correlation between parameters. *P*-values smaller than 5% were considered to be significant.

### Results

### *Fluorescence ratio imaging in thick samples: the PIOS scheme*

To improve fluorescence measurements in thick samples, an optical sectioning effect (or partial confocal effect) was created by introducing pinhole diaphragms of different diameters in the path of the excitation light beam (Figure 1).

First, the depth-of-light collection was determined *in vitro*. A sodium phosphate buffer solution was placed in cuvettes with increasing depths (0 to 800 $\mu$m), with a fixed concentration (0.002 mg $ml^{-1}$) of the BCECF probe. Wide-field illumination (i.e. no diaphragm in the excitation path) resulted in a steady increase in fluorescence intensities with increasing depths (0–800 $\mu$m), both at 440 and 495 nm; it evidenced the contamination of the in-focus fluorescence by out-of-focus haze. In contrast, the use of a 40 $\mu$m pinhole in the excitation light path limited the depth-of-light collection to 40 $\mu$m at both wavelengths (Figure 2). Importantly, fluorescence ratios, as determined by equation (1), were stable at any depth $>40$ $\mu$m using a 40 $\mu$m pinhole, whereas they decreased drastically with increasing depths under wide-field excitation (data not shown).

The effects of this pinhole configuration on fluorescence ratios were investigated further by progressively decreasing the size of the illumination spot (pinhole diaphragm) focused on the fluorescent sample. At a fixed cuvette depth (800 $\mu$m), fluorescence ratios decreased with decreasing apertures, until a minimum ratio was reached with the 40 $\mu$m pinhole. A further reduction in the aperture (20 $\mu$m pinhole) did not significantly affect the ratio. The same study was performed *in vivo*, in a 2-mm-thick LS174T tumour grown in the dorsal skinfold chamber. Results were similar to the *in vitro* case, namely a stable fluorescence ratio with apertures less than or equal to 40 $\mu$m (data not shown). Thus, for subsequent *in vivo* studies, the 40 $\mu$m pinhole aperture was used, since it provided stable ratios, while maintaining a sufficiently high fluorescence emission signal.

**Figure 2** Fluorescence intensity at various cuvette depths *in vitro*. Excitation wavelengths were 440 nm (●) and 495 nm (▲). AU, arbitrary units. Mean $\pm$ s.e.m. is shown ($n=3$).

*Calibration*

*In vitro* calibration (see Materials and methods) was performed over a wide pH range (4.51–9.25), using the pinhole configuration on the microscope. As expected, a sigmoidal relationship between pH and the measured fluorescence ratio at 495 and 440 nm was obtained (insert, Figure 3). In the 6.20–7.80 pH range, the relationship was well approximated by a linear fit ($r^2=0.99$; open symbols, Figure 3). *Ex vivo* calibration of excised normal (subcutaneous) and tumour tissues again revealed a linear relationship ($r^2=0.98$ in both cases; closed symbols, Figure 3) between actual pH and measured ratios, in the pH range of interest for subsequent *in vivo* studies (6.20–7.40). Different slopes were observed between the two *ex vivo* curves; these reflect intrinsic spectroscopic differences in tumour *vs* normal tissues. Accordingly, when reporting tumour *vs* normal tissue data *in vivo*, the corresponding *ex vivo* calibration curves were used.

*Tumour interstitial pH is lower than normal subcutaneous pH*

The modified FRIM technique was used to measure interstitial pH in the 21-day-old, LS174T human colon adenocarcinoma xenograft ($N=6$ animals) and in normal subcutaneous tissue ($N=6$). Tumour interstitial pH was $6.96\pm0.03$ ($\pm$s.e.m.), which was significantly lower than the pH of subcutaneous tissue ($7.37\pm0.09$).

*Interstitial pH gradients*

The application of FRIM to thick tissues *in vivo* using the PIOS modification made it possible to obtain local interstitial pH profiles in three-dimensional solid tumours. Figure 4a depicts two tumour blood vessels, 38 μm (left) and 29 μm (right) in diameter, separated by a distance of 120 μm. Figure 4b shows the corresponding interstitial pH profile between the two vessels. A definite pH gradient, starting from either vessel, was observed. Highest pH ($pH_{max}=7.44$) was recorded 10 μm away from the vessels; pH then gradually decreased to reach a minimum ($pH_{min}=7.15$; $\Delta pH=pH_{max}-pH_{min}=0.29$) around the midpoint of the intervessel distance.

Ratio measurements less than 10 μm from a blood vessel were not valid owing to differential light absorption by blood haemoglobin at 440 and 495 nm. This always resulted in an uncontrollable distortion of the ratio signal, within the vessel and in its proximity (<10 μm). Also, when choosing tumour locations at the beginning of each experiment, it was checked, by focusing the microscope through the sampling volume, that no underlying blood vessels were crossing the in-focus interstitial area of interest. The possible presence of an 'invisible' vessel during the interstitial scan was readily identified during offline analysis by spurious jumps in the ratio signal (2- to 5-fold increase in ratio); such profiles were discarded from subsequent analyses. To confirm these observations, we performed control experiments *in vitro* using saline solutions mixed with mouse blood at different haematocrits ($H_T$), at pH = 7.2. Compared with the pH ratio of a control saline solution (no RBC), the ratios of solutions adjusted to normal mouse arterial blood $H_T$, 1/10 normal $H_T$, and 1/100 $H_T$ showed 9.0-fold, 7.5-fold, and 2.2-fold increases respectively.

For a given intervessel distance, interstitial pH gradients could exhibit different shapes, such as: (1) a parabolic shape, as shown in Figure 4b, with a local minimum around the midpoint of the intervessel distance (7 out of 17 profiles); (2) a skewed parabolic shape, with the local minimum being closer to one of the two vessels (7/17); or (3) a 'cuvette' shape (Figure 4c), characterised by an initial steep pH decrease from both vessel sides and a low, flat interstitial pH profile in between (3/17).

The relationship between the intervessel distance and the maximal pH drop, ΔpH, was studied. ΔpH was defined as the difference between $pH_{max}$, the pH measured 10 μm from one of the two blood vessels, and $pH_{min}$, the minimum pH measured between the two vessels. Blood vessels with comparable diameters ($20\pm3$ μm, mean±s.e.m.), RBC velocities ($0.15\pm0.02$ mm $s^{-1}$) and hence flow rates were selected. Figure 4d shows that ΔpH significantly increased with increasing intervessel distances (Spearman correlation coefficient, $r_s=0.723$; $P<0.001$).

**Figure 3** Calibration of fluorescence ratios (see Materials and methods). □, *In vitro*; linear fit: $y=-5.63+0.98x$. ■, *Ex vivo*, using tumour tissue; linear fit: $y=-7.68+1.28x$. ▲, *Ex vivo*, using normal tissue; linear fit: $y=-4.66+0.81x$. Insert: full scale of the *in vitro* calibration. Mean±s.e.m. is shown ($n=5$).

*Glucose-induced modification of pH gradients in solid tumours*

In any given tumour location, a bolus injection of glucose (6 g $kg^{-1}$ i.v.) induced a decrease in pH of at least 0.1 pH unit (31 locations in ten tumours). Hyperglycaemia did not significantly change interstitial pH of normal subcutaneous tissue (data not shown). The effect of glucose on tumour interstitial pH gradients is shown in detail in Figure 5. Forty-five minutes after glucose treatment, interstitial pH monotonically decreased with increasing distance from the blood vessel, resulting in a pH gradient which was very similar in shape (i.e. no significant changes in slope), but significantly lower, compared with the gradient observed *before* glucose injection. Ninety minutes after glucose administration, the pH gradient had returned to near-baseline values (Figure 5). When compared with pretreatment values, local blood flow (in the vessel adjacent to the area of pH measurement) was not affected, 45 and 90 min after glucose injection. This was evidenced by no significant changes in the measured RBC velocities and vessel diameters (Table I) and in the corresponding flow rates.

*Temporal dynamics and intratumour heterogeneity of glucose-induced pH changes*

Figure 6a is a low-magnification, transillumination image of a 21-day-old LS174T tumour xenograft. Heterogeneous vascularisation is evidenced by vessel-rich areas (e.g. labels #3, 4 and 5), areas totally devoid of blood vessels (e.g. label #1), and areas at the interface between the two (e.g. labels #2 and 6). Using the PIOS-modified fluorescence ratio imaging technique, we measured temporal pH dynamics in such interstitial areas, before and after glucose treatments.

**Figure 4** (**a**) Transillumination image, at the microcirculatory level, of a LS174T tumour xenograft grown in the dorsal skinfold chamber (20 × objective). (**b**) Interstitial pH profile along the line shown in **a**. The two blood vessels seen in **a** are located at the 0 and 120 $\mu$m abscissae. (**c**) Interstitial pH profile in another tumour location. Vessels were 140 $\mu$m apart (located at the 0 and 140 $\mu$m abscissae). (**d**) The effect of the intervessel distance on ΔpH, the maximal pH drop (see text). Spearman correlation coefficient $r_s = 0.723$; $P < 0.001$.

**Figure 5** Interstitial pH as a function of distance from the blood vessel. ○, before glucose; □, 45 min after glucose injection (6 g kg$^{-1}$ i.v.); ■, 90 min after glucose. Data were averaged over a total of $n=13$ locations in 6 tumours. Mean ± s.e.m. is shown.

**Table I** Vessel diameters and RBC velocities adjacent to the interstitial areas chosen for pH measurements before and after glucose infection

| | *Baseline (0 min)* | *45 min* | *90 min* |
|---|---|---|---|
| Vessel diameter (μm)[a] | 23±5 | 22±8 | 28±5 |
| RBC velocity (mm s$^{-1}$)[a] | 0.18±0.01 | 0.17±0.02 | 0.18±0.01 |

[a]$N=6$ animals, $n=13$ measurements; mean ± s.e.m.

Figure 6b shows that baseline pH, while remaining relatively stable within a 25 min period, could be extremely heterogeneous among different tumour locations. Lowest pH was always found within or near avascularised areas (curves #1, 2 and 6 in Figure 6b, which match the labels in Figure 6a). Figure 6b further shows that glucose induced a pH decrease in all areas. However, the temporal characteristics differed, with an earlier and/or sharper pH drop in vascularised areas (curves 4 and 5, Figure 6b), as compared with non-vascularised areas (curves 1, 2 and 6, Figure 6b). This was confirmed by averaging the data over several such locations in $N=5$ tumours. In well-vascularised areas, a significant pH drop of nearly 0.15 pH unit was evident 20 min after glucose injection (closed symbols, Figure 6c). In non-vascularised areas, pH decrease was more progressive and significantly dropped by ≈0.15 pH unit after 60 min (open symbols, Figure 6c), a time point at which pH was already returning to baseline values in vascularised areas.

### *The effect of sodium bicarbonate treatment on pH in solid tumours*

Chronic ingestion of 1% sodium bicarbonate (see Materials and methods) tended to decrease interstitial pH in solid tumours; however, differences were not statistically significant when compared with controls (Table II). This was true whether measurements were performed within densely vascularised or avascular regions. Similarly, an acute sodium bicarbonate treatment (see Materials and methods) did not induce significant pH changes (data not shown).

### Discussion

### *Interstitial pH gradients*

Intervessel pH gradients were detected in a solid tumour using the modified FRIM technique (Figure 4b). The average pH decrease, away from the blood vessel (0.14 units at 50 μm; Figure 5) was similar to that reported by Martin and Jain (1994) for a thin tumour model (0.13 units at 50 μm). However, in the latter case, the average pH gradient, 30 μm away from the blood vessel, was nearly zero; in the present study, pH kept decreasing monotonically in the 10 to 70 μm range. This may be expected from our three-dimensional experimental tumours, where acid accumulation in the full thickness of the tissue is possible. The gradient data (Figure 5) confirm theoretical predictions made for solid tumours (Von Ardenne and Von Ardenne, 1977) and tumour spheroids (Casciari *et al*., 1992*a*). In particular, Von Ardenne and Von Ardenne (1977) predicted a pH drop of 7.2 to 6.9 (0.3 pH units) over a 60 μm distance from the capillary wall into the tumour tissue, while we report here a 0.33 unit decrease over a 70 μm distance (Figure 5).

The parabolic pH profile (Figure 4b) between vessels could be explained by corresponding gradients in $pO_2$, due to limitations in oxygen penetration. Thus, the percentage of tumour cells that rely solely on glycolysis for energy production would increase with distance from the blood vessel. In support of this, previous studies have shown: (1) the existence of $pO_2$ gradients away from the blood vessel in solid tumours (Dewhirst *et al*., 1994; Torres-Filho *et al*., 1994); a decrease of both $pO_2$ and pH at increasing depths in spheroids and some rodent tumours (Kallinowski and Vaupel, 1988; Vaupel *et al*., 1981); and (2) an increased glucose consumption rate by tumour spheroids with decreased oxygen concentration (Casciari *et al*., 1992*b*). pH gradients could be further steepened by poor removal of metabolic waste products, owing to inadequate vascularisation and/or insufficient local blood flow (Jain, 1988). Enhanced glycolysis (Casciari *et al*., 1992*b*), higher $pCO_2$ and dissolved carbon dioxide levels (Gullino *et al*., 1965), and lower bicarbonate concentration (Gullino *et al*., 1965) in the interstitium would then all contribute to decreasing pH values away from the blood vessel.

Interestingly, not all pH profiles were symmetric about the midpoint of the intervessel distance. Skewed parabolic shapes, with the local minimum being closer to one of the two vessels, were measured. Such profiles could be explained by differential blood flow between the two surrounding vessels, and, subsequently, differential nutrient delivery and metabolic waste removal rates. Heterogeneity of metabolic rates among tumour cells may also be a factor. Alternatively, asymmetric profiles could be generated by underlying blood vessels, at a depth where they would not contribute to the ratio signal (see Results), but affect the metabolism of the overlying tissue through diffusion of nutrients and/or metabolites. pH profiles exhibiting a 'cuvette' shape (i.e. an initial steep pH decrease from each vessel side and a low flat pH profile in between) were also measured (Figure 4c). This pH plateauing could reflect limits in glucose consumption and glycolysis rates at low extracellular pH. Such an inhibition of the Crabtree-like effect has been reported earlier in: (1) tumour cells *in vitro*, where a 38% decrease in glucose consumption rate was induced when pH was reduced from 7.0 to 6.5 (Rotin *et al*., 1986); and (2) tumour spheroids, where the glucose consumption rate decreased by ≈60% when pH was changed from 7.25 to 6.95 (Casciari *et al*., 1992*b*). In addition, lactate transport out of the cell is known to be inhibited at low extracellular pH *in vitro* (Spencer and Lehninger, 1976), which would further contribute to this plateauing effect.

A significant, positive correlation was found between the maximal pH drop (from the vessel to the intervessel

**Figure 6** (**a**) Transillumination image of a LS174T tumour xenograft (bar = 1 mm). (**b**) Glucose-induced pH changes in six different tumour locations (curves with open symbols; numbers correspond to labels shown in **a**). Closed symbols and bold-line fit, average of the six locations. (**c**) Glucose-induced pH changes in well-vascularised tumour areas (▲; $n = 12$ in five tumours), non-vascularised areas (◆; $n = 6$ in five tumours), and at the interface of vascularised/non-vascularised areas (□; $n = 6$ in five tumours). Mean ± s.e.m. is shown.

**Table II** Effect of chronic sodium bicarbonate ingestion on interstitial tumour pH

| | *pH (mean ± s.e.m.) controls* | *pH (mean ± s.e.m.) after treatment (8 days 1% sodium bicarbonate in drinking water* |
|---|---|---|
| Vascular areas | 7.04 ± 0.03 ($n$ = 15, $N$ = 6)[a] | 6.93 ± 0.21 ($n$ = 15, $N$ = 6) |
| Avascular areas | 6.78 ± 0.05 ($n$ = 7, $N$ = 3) | 6.67 ± 0.80 ($n$ = 4, $N$ = 3) |

[a] $N$, number of animals; $n$, total number of measurements.

midpoint) and the intervessel distance; however, large data scatter was observed (Figure 4d). This is expected, since interstitial pH will depend markedly on the local degree of vascularisation, network arrangement of vessels, and tumour blood flow, all of which are known to be largely heterogeneous in tumours (Jain, 1988; Vaupel *et al.*, 1989). It is further supported by the finding that baseline pH values are lower (6.78 ± 0.04) in avascular regions, intermediate (6.88 ± 0.05) at the interface of avascular/vascular regions, and higher (7.04 ± 0.03) in densely vascularised regions (Figure 6a–c, before glucose treatment).

*Glucose-induced modulation of interstitial pH and pH gradients*

A bolus i.v. injection of glucose induced a pH decrease in all tumour areas investigated, while it had no effect on normal subcutaneous tissue pH. This is in agreement with the abundant literature on selective tumour acidification by hyperglycaemia [for a review see Ward and Jain (1988)]. The glucose-induced pH decreases in the LS174T tumours (maximum drop of 0.10–0.15 pH units < 60 min, Figure 6c) were smaller and more rapid than those reported by Martin and Jain (1994) (drop of 0.30 units after 120 min), Volk *et al.* (1993) (drop of 0.40 units at 2–4 h), and Gerweck *et al.* (1991) (drop of 1.13 units at 2–3 h). The response to glucose, however depends strongly on the tumour model, dose, time schedule and route of administration (Ward and Jain, 1988).

We further show that the temporal dynamics of glucose-induced pH decrease strongly depend on tumour location and vascularisation (Figure 6). Although the pH drop following a bolus glucose injection was transient in all cases (Figure 6b), the response and recovery times were faster, and most importantly, the maximum pH drop occurred earlier, when comparing densely vascularised to avascular areas (Figure 6c). This may be directly related to the blood flow-dependent availability of the glucose substrate (Kallinowski *et al.*, 1988). Interestingly, averaging the data over different locations within a tumour at different time points (Figure 6b, bold line) resulted in a glucose-induced pH response which could be either similar to (e.g. Figure 6, curve 3), or significantly different from (e.g. Figure 6, curves 1 and 5) the response of individual locations. Such results should help to sort out part of the heterogeneity reported on this subject in the literature, which may not be solely due to differences in experimental protocols (Ward and Jain, 1988).

The interstitial pH profile was shifted to lower pH values (0.15 units) after glucose injection (45 min), when compared with pretreatment values. However, the gradient was not significantly altered (Figure 5) at this 45 min time point (it may have differed at earlier time points). Thus, within the distance of 10 to 70 μm from the blood vessel, there could be a balance between glucose delivery (and consumption, assuming all delivered glucose is metabolised) and metabolic (acidic) waste production. Proximal to the blood vessel, high glucose delivery/metabolism, $H^+$ production and removal can be expected, resulting in a pH drop of 0.15 units; distally, reduced glucose delivery, owing to proximal consumption and possible diffusion limitations (Casciari *et al.*, 1988), accompanied by reduced $H^+$ production and removal, may result in a similar pH drop (0.15 units). These data are also consistent with the finding that tumour metabolism is mainly determined by the availability of the glucose substrate, rather than by the metabolic demand of the cancer cells (Kallinowski *et al.*, 1988).

Also, we found no significant effect on the local blood flow rate in the vessel adjacent to the measurement area (as assessed by RBC velocities and vessel diameters, Table I), 45 and 90 min after glucose injection. Hyperglycaemia is known to decrease the mean tumour blood flow rate [as averaged for several vessels within a tumour (Ward-Hartley and Jain, 1987), or as measured for the whole tumour (DiPette *et al.*, 1986)] at similar time points in different animal models. However, the present study shows that such a decrease in flow does not necessarily occur in every single tumour blood vessel; this is also consistent with the well-known spatial heterogeneity in tumour blood flow (Jain, 1988). Also, we cannot exclude a more dramatic hyperglycaemic effect on tumour blood flow at earlier time points (< 45 min).

*Implications of spatially and temporally modulated pH for cancer therapy*

Tumour pH has been proposed as a prognostic factor for cancer therapy (Engin *et al.*, 1994; Gatenby, 1995; Van den Berg *et al.*, 1992). The present study shows that low pH values (< 6.8) exist locally. Furthermore, pH is spatially modulated and strongly depends on the local vascularisation. Therefore, averaged pH values or a few local pH measurements may not be adequate as a prognostic factor.

Steep interstitial pH gradients, as reported here, with pH values ranging from 6.5 to 7.4, will also affect the outcome of various chemotherapy modalities. Tumour cytotoxic alkylating agents, whose cellular uptake is favoured at low extracellular pH, will be more effective distally from functional vessels (assuming effective tissue penetration). Similarly, cytotoxic intracellular acidification caused by weak acids (e.g. succinate, malonate $4 < pK_a < 6$) *in vitro* is effective at low extracellular pH (< 6.5), but not at neutral pH (Karuri *et al.*, 1993).

The glucose data support the idea that hyperglycaemia can be used as an adjuvant treatment to decrease interstitial tumour pH acutely and selectively. This would be particularly useful in therapies, such as hyperthermia, where tumour cells which are chronically exposed to low pH become treatment resistant. However, differences in the spatial and temporal courses of the response exist and must be considered carefully (Figure 6c). In contrast, the present study (Table II), as well as the data of Jähde and Rajewsky (1982), do not confirm the initial report on selective tumour acidification induced by sodium bicarbonate ingestion (Gullino *et al.*, 1965). Thus, selective bicarbonate-induced decrease in interstitial tumour pH appears to be more species- and/or tumour-specific than hyperglycaemia-induced tumour acidification.

*Advantages and limitations of the PIOS–FRIM technique*

In the wide-field illumination mode or with any diaphragm aperture > 40 μm, stable fluorescence ratios could not be obtained *in vitro*. When applied *in vivo*, the situation worsened, owing to significant light scattering by the thick tissue sample. During the imaging process, scattering was evidenced by a halo of fluorescence emission light (excluded from the analysis), which was imaged outside the original excitation spot; it could extend > 1 spot diameter beyond the spot area and strongly depended on the tissue location (not shown). As a result, fluorescence ratios obtained in the wide-field mode were unstable, even under baseline conditions. Another source of error in the wide-field mode came from the possible presence of unseen underlying blood vessels. These would certainly distort the ratio signal, since: (1) light absorption by haemoglobin is different at the 440 *vs* 495 nm wavelengths; and (2) wide-field light collection *in vitro* extended to 800 μm depths. By introducing a 40 μm pinhole to limit the size of the excitation light spot, contamination of the emission image by scattered light and differential light absorption was minimised. The trade-off was an increase in acquisition time and storage space; thus, a gradient measurement necessitated scanning of the excitation spot along the chosen gradient line. However, time resolution was still adequate for the pH dynamics reported here.

Also, at physiological interstitial pH (7.2–7.4), the BCECF probe, with a $pK_a$ of 6.98, is mostly in its ionised (non-protonated) form, with four to five negative charges. However, as interstitial tumour pH decreases to more acidic values (<7.0), an increasing fraction of the probe will become protonated and may permeate the tumour cell membrane. Thus, at low interstitial pH (<7.0), intracellular fluorescence may contribute to the extracellular pH signal reported in this study. We believe this effect is negligible for the following reasons: (1) BCECF has different $pK_a$ values for each of its ionising groups, and the entry of BCECF into cells would depend upon protonation of all of the charged groups, a rare event at pH values reported in this study; (2) our measurements using the cell-impermeable probe BCECF-dextran (MW 10 000, 80 mg $kg^{-1}$ i.v.; Molecular Probes) resulted in pH values virtually identical to those obtained with BCECF-acid (data not shown); and (3) rapid interstitial clearance of the BCECF-acid probe (<1 h; data not shown) argues against intracellular trapping.

In the future, the FRIM technique may be further adapted and used in combination with a phosphorescence quenching technique (Torres-Filho *et al.*, 1994), to evaluate non-invasively extracellular pH, intracellular pH and $pO_2$ in solid tumours transplanted in the dorsal chambers in mice. Using glycolysis-deficient cells (Pouysségur *et al.*, 1980), the simultaneous knowledge of pH/$pO_2$ may help determine the origin of non-glycolytic acidification, as reported in some tumours (Hwang *et al.*, 1992; Newell *et al.*, 1993).

### Abbreviations

BCECF, 2′,7′-bis-(2-carboxyethyl)-5,6-carboxyfluorescein; FRIM, fluorescence ratio imaging; PIOS; pinhole illumination and optical sectioning; RBC, red blood cell; SCID, severe combined immunodeficient.

### Acknowledgements

This work was supported by a NCI Outstanding Investigator Grant (R35-CA-56591) to RK Jain. M Dellian is a recipient of a Feodor-Lynen Fellowship from the Alexander von Humboldt Foundation (1993–1995). The authors thank DA Berk and LE Gerweck for their helpful advice.

### References

ASHBY B. (1966). pH studies in human malignant tumours. *Lancet*, **2**, 312–315.

BRIGHT GR. (1993). Fluorescence ratio imaging: issues and artefacts. In *Optical Microscopy: Emerging Methods and Applications*, Herman B and Lemasters LL. (eds), pp. 87–114. Academic Press: New York.

CASCIARI J, SOTIRCHOS S AND SUTHERLAND R. (1988). Glucose diffusivity in multicellular tumor spheroids. *Cancer Res.*, **48**, 3905–3909.

CASCIARI J, SOTIRCHOS S AND SUTHERLAND R. (1992*a*). Mathematical modeling of microenvironment and growth in EMT6/Ro multicellular tumour spheroids. *Cell Prolif.*, **25**, 1–22.

CASCIARI J, SOTIRCHOS S AND SUTHERLAND R. (1992*b*). Variations in tumor cell growth rates and metabolism with oxygen concentration, glucose concentration, and extracellular pH. *J. Cell. Physiol.*, **151**, 386–394.

DALY P AND COHEN J. (1989). Magnetic resonance spectroscopy of tumors and potential *in vivo* clinical applications: a review. *Cancer Res.*, **49**, 770–779.

DEWHIRST M, SECOMB T, ONG E, HSU R AND GROSS J. (1994). Determination of local oxygen consumption rates in tumors. *Cancer Res.*, **54**, 3333–3336.

DIPETTE DJ, WARD-HARTLEY KA AND JAIN RK. (1986). Effect of glucose on systemic hemodynamics and blood flow rate in normal and tumor tissues in rats. *Cancer Res.*, **46**, 6299–6304.

ENGIN K, LEEPER D, THISTLETHWAITE A, TUPCHONG L AND MCFARLANE J. (1994). Tumor extracellular pH as a prognostic factor in thermoradiotherapy. *Int. J. Radiat. Oncol. Biol. Phys.* **29**, 125–132.

GATENBY R. (1995). The potential role of transformation-induced metabolic changes in tumor–host interaction. *Cancer Res.*, **55**, 4151–4156.

GERWECK L, RHEE J, KOUTCHER J, SONG C AND URANO M. (1991). Regulation of pH in murine tumor and muscle. *Radiat. Res.*, **126**, 206–209.

GULLINO P, GRANTHAM F, SMITH S AND HAGGERTY A. (1965). Modifications of the acid-base status of the internal milieu of tumors. *J. Natl Cancer Inst.*, **34**, 857–869.

HAWKINS R AND PHELPS M. (1988). PET in clinical oncology. *Cancer Met. Rev.*, **7**, 119–142.

HWANG Y, KIM S-G, EVELHOCH J AND ACKERMAN J. (1992). Nonglycolytic acidification of murine radiation-induced fibrosarcoma 1 tumor via 3-O-Methyl-D-glucose monitored by $^{1}H$, $^{2}H$, $^{13}C$, and $^{31}P$ nuclear magnetic resonance spectroscopy. *Cancer Res.*, **52**, 1259–1266.

JÄHDE E AND RAJEWSKY M. (1982). Tumor-selective modification of cellular microenvironment *in vivo*; effect of glucose infusion on the pH in normal and malignant rat tissues. *Cancer Res.*, **42**, 1505–1512.

JAIN RK. (1988). Determinants of tumor blood flow: a review. *Cancer Res.*, **48**, 2641–2658.

KALLINOWSKI F AND VAUPEL P. (1988). pH distribution in spontaneous and isotransplanted rat tumours. *Br. J. Cancer*, **58**, 314–321.

KALLINOWSKI F, VAUPEL P, RUNKEL S, BERG G, FORTMEYER H, BAESSLER K, WAGNER K, MUELLER-KLIESER W AND WALENTA S. (1988). Glucose uptake, lactate release, ketone body turnover, metabolic micromilieu, and pH distributions in human breast cancer xenografts in nude rats. *Cancer Res.*, **48**, 7264–7272.

KANEKO K, GUTH P AND KAUNITZ J. (1991). *In vivo* measurement of rat gastric surface cell intracellular pH. *Am. J. Physiol.*, **261**, G548–G552.

KARURI A, DOBROWSKY E AND TANNOCK I. (1993). Selective acidification and toxicity of weak organic acids in an acidic microenvironment. *Br. J. Cancer*, **68**, 1080–1087.

LEUNIG M, YUAN F, MENGER MD, BOUCHER Y, GOETZ AE, MESSMER K AND JAIN RK. (1992). Angiogenesis, microvascular architecture, microhemodynamics, and interstitial fluid pressure during early growth of human adenocarcinoma LS174T in SCID mice. *Cancer Res.*, **52**, 6553–6560.

MARTIN GR AND JAIN RK. (1993). Fluorescence ratio imaging measurement of pH gradients: calibration and application in normal and tumor tissues. *Microvasc Res.*, **46**, 216–230.

MARTIN GR AND JAIN RK. (1994). Noninvasive measurement of interstitial pH profiles in normal and neoplastic tissue using fluorescence ratio imaging microscopy. *Cancer Res.*, **54**, 5670–5674.

MORDON S, DEVOISSELLE J AND MAUNOURY V. (1994). *In vivo* pH measurement and imaging of tumor tissue using a pH-sensitive fluorescent probe (5,6-carboxyfluorescein):instrumental and experimental studies. *Photochem. Photobiol.*, **60**, 274–279.

NEWELL K, FRANCHI A, POUYSSÉGUR J AND TANNOCK I. (1993). Studies with glycolysis-deficient cells suggest that production of lactic acid is not the only cause of tumor acidity. *Proc. Natl Acad. Sci. USA*, **90**, 1127–1131.

PITTMAN R. (1986). *In vivo* photometric analysis of hemoglobin. *Ann. Biomed. Eng.*, **14**, 119–137.

POUYSSÉGUR J, FRANCHI A, SALOMON J AND SILVESTRE P. (1980). Isolation of a Chinese hamster fibroblast mutant defective in hexose transport and aerobic glycolysis: its use to dissect the malignant phenotype. *Proc. Natl Acad. Sci. USA*, **77**, 2698–2701.

ROTIN D, ROBINSON B AND TANNOCK I. (1986). Influence of hypoxia and acid environment on the metabolism and viability of cultured cells: potential applications of cell death in tumors. *Cancer Res.*, **46**, 2812–2826.

SONG C, LYONS J AND LUO Y. (1993). Intra- and extracellular pH in solid tumors: influence on therapeutic response. In *Drug Resistance in Oncology*, Teicher B. (ed) pp. 25–51. Marcel Dekker: New York.

SPENCER T AND LEHNINGER A. (1976). L-lactate transport in Ehrlich ascites tumour cells. *Biochem. J.*, **154**, 405–414.

TANKE H, VAN OOSTVELDT P AND VAN DUIJN P. (1982). A parameter for the distribution of fluorophores in cells derived from measurements of innerfilter effect and reabsorption phenomenon. *Cytometry*, **6**, 359–369.

TORRES-FILHO IP, LEUNIG M, YUAN F, INTAGLIETTA M AND JAIN RK. (1994). Noninvasive measurement of microvascular and interstitial oxygen profiles in a human tumor in SCID mice. *Proc. Natl Acad. Sci. USA*, **91**, 2081–2085.

VAN DEN BERG A, VAN DE MERWE S AND VAN DER ZEE J. (1992). Prognostic value of tumor tissue pH for tumor response to hyperthermia. In *Radiation Research: A Twentieth Century Perspective*, Dewey W, Edington M, Fry R, Hall E and Whitmore G. (eds), Vol. 2. pp. 951–956, Taylor A Francis: London.

VAN OOSTVELDT P AND BAUWENS S. (1990). Quantitative fluorescence in confocal microscopy. *J. Micros.*, **158**, 121–132.

VAUPEL P, FRINAK S AND BICHER H. (1981). Heterogeneous oxygen partial pressure and pH distribution in C3H mouse mammary adenocarcinoma. *Cancer Res.*, **41**, 2008–2013.

VAUPEL P, KALLINOWSKI F AND OKUNIEFF P. (1989). Blood flow, oxygen and nutrient supply, and metabolic microenvironment of human tumors: a review. *Cancer Res.*, **49**, 6449–6465.

VOLK T, JÄHDE E, FORTMEYER H, GLÜSENKAMP K-H AND RAJEWSKY M. (1993). pH in human tumour xenografts: effect of intravenous administration of glucose. *Br. J. Cancer*, **68**, 494–500.

VON ARDENNE M AND VON ARDENNE A. (1977). Berechnung des pH-Profils im Interkapillarraum der Krebsgewebe für die Fälle mit und ohne Langzeit-Glukose-Infusion. *Res. Exp. Med.*, **171**, 177–189.

WARD KA AND JAIN RK. (1988). Response of tumours to hyperglycaemia: characterization, significance and role in hyperthermia. *Int. J. Hyperthermia*, **4**, 223–250.

WARD-HARTLEY KA AND JAIN RK. (1987). Effect of glucose and galactose on microcirculatory flow in normal and neoplastic tissues in rabbits. *Cancer Res.*, **47**, 371–377.


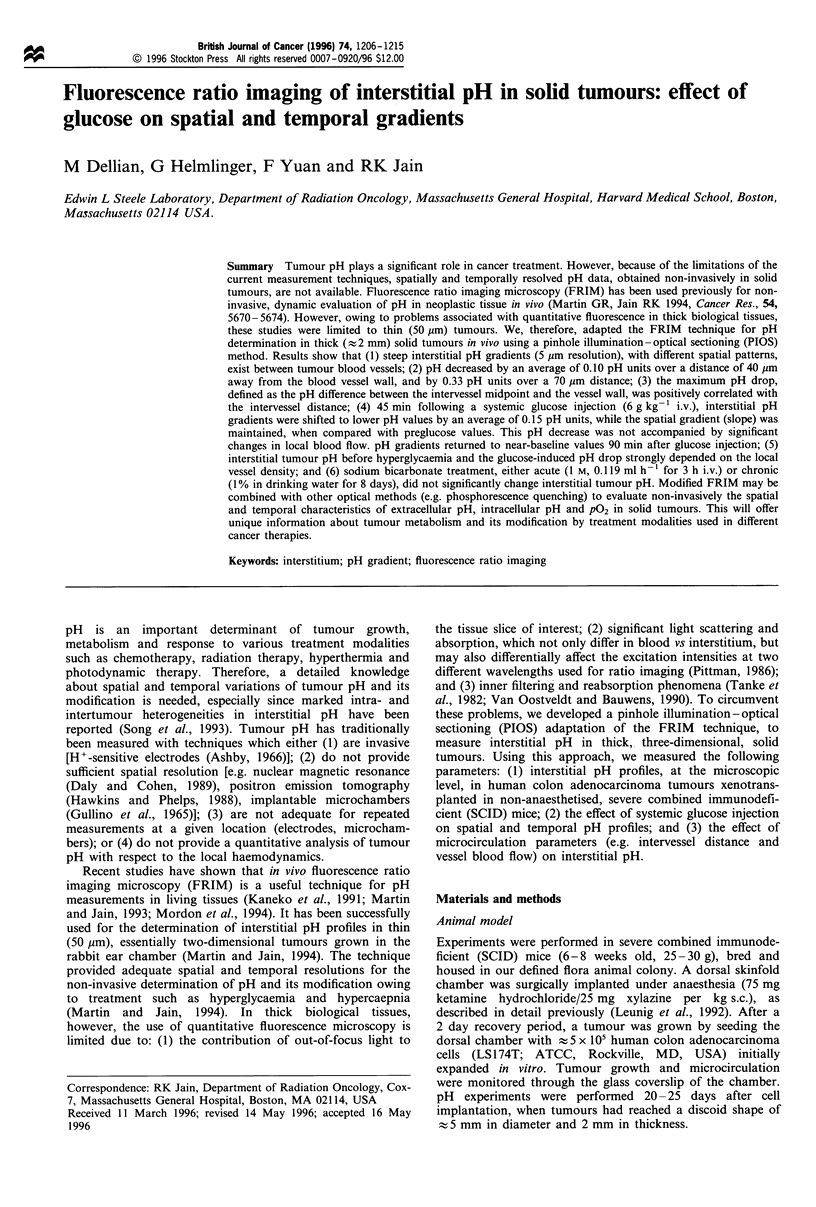

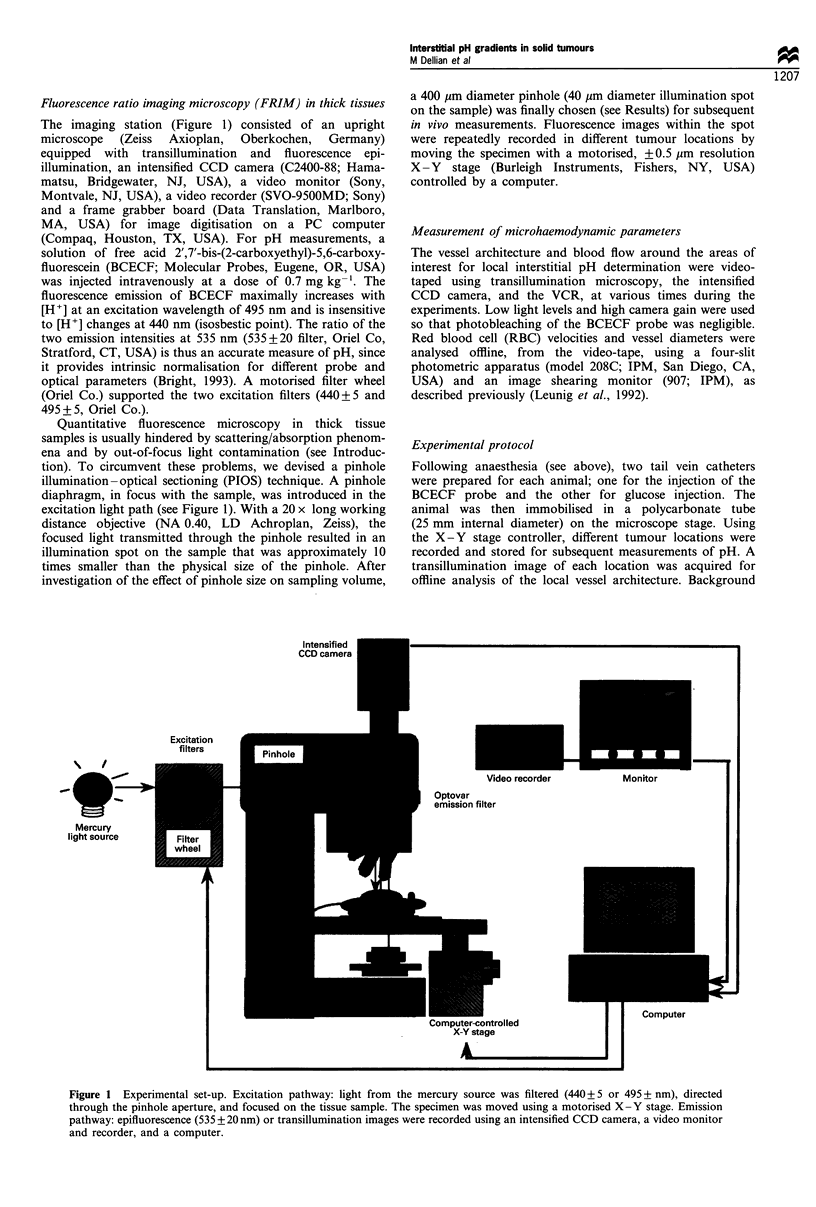

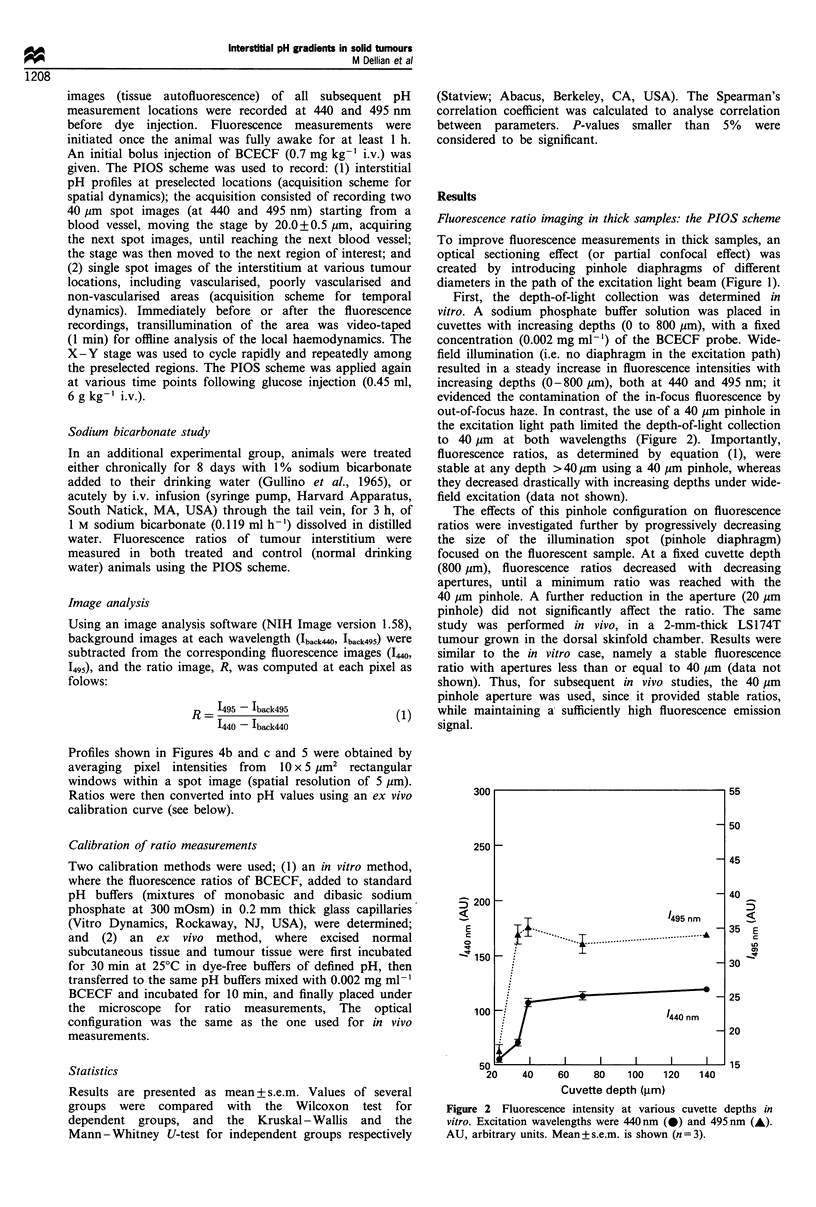

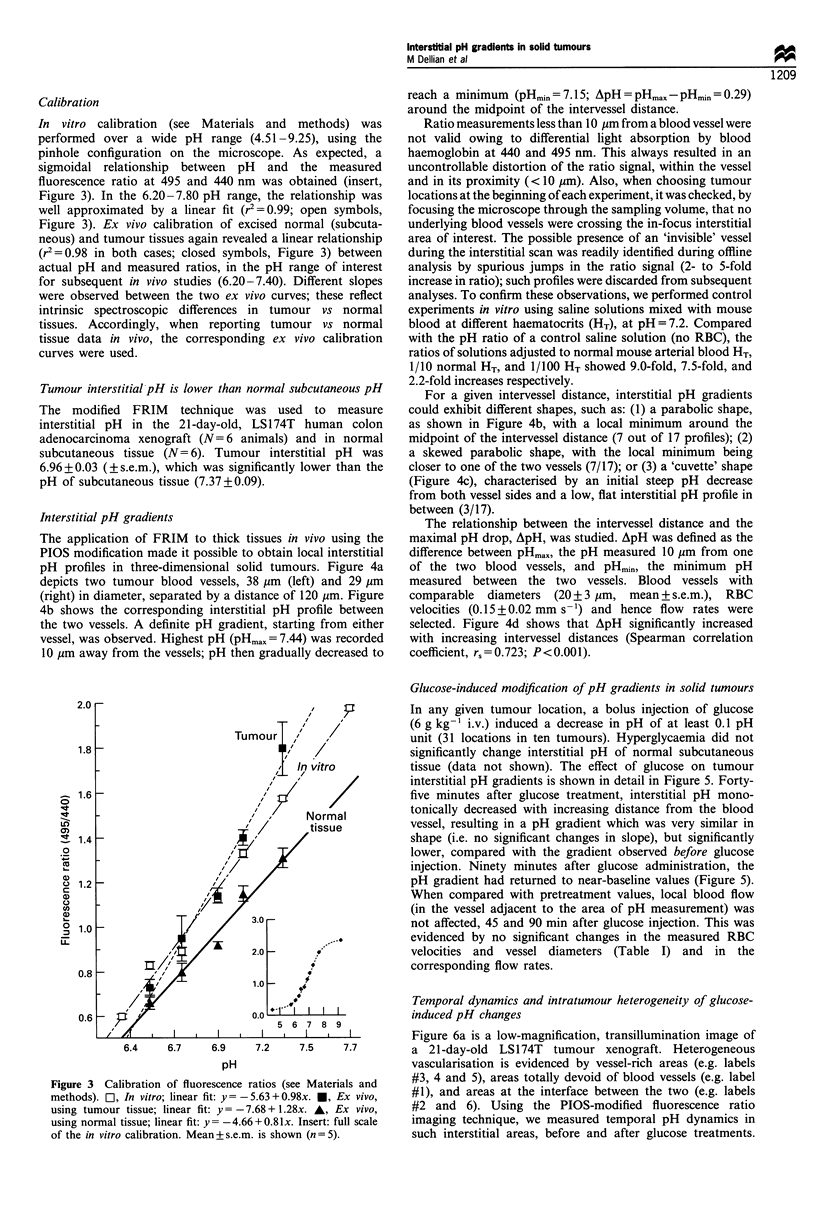

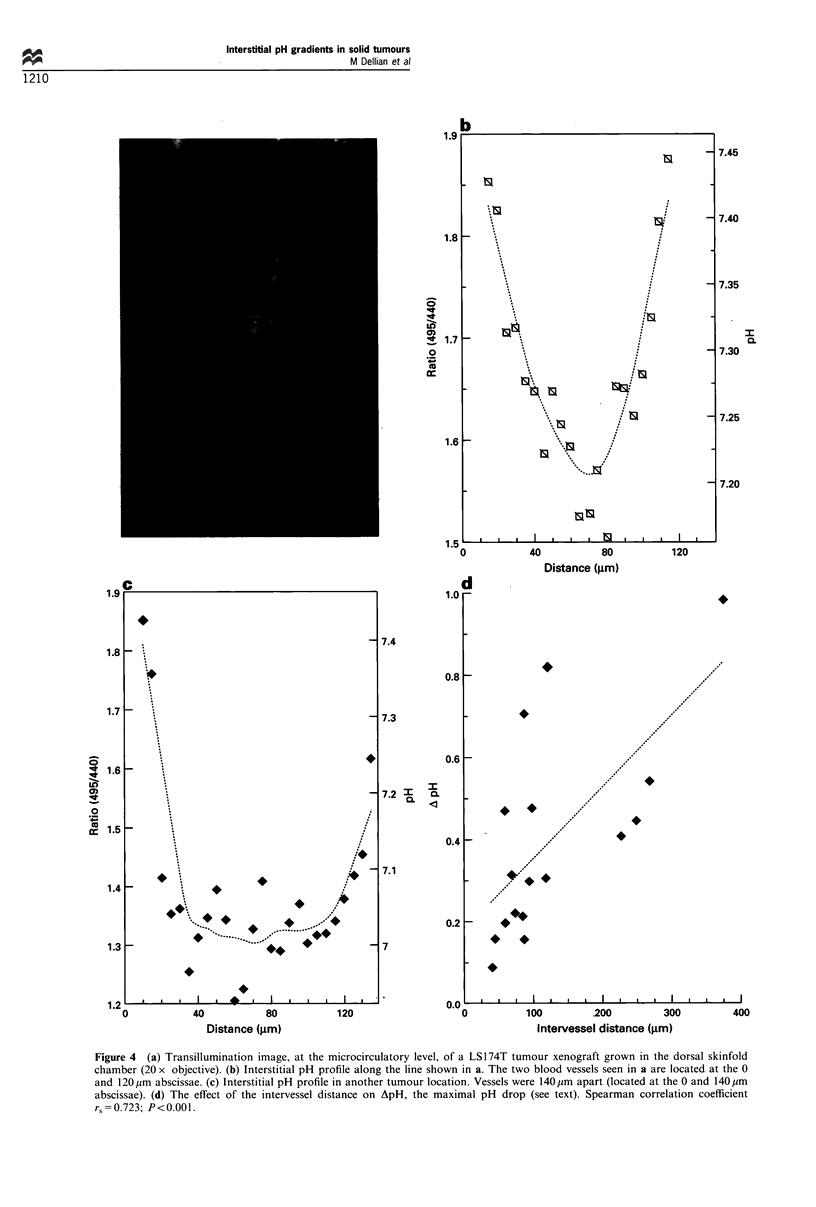

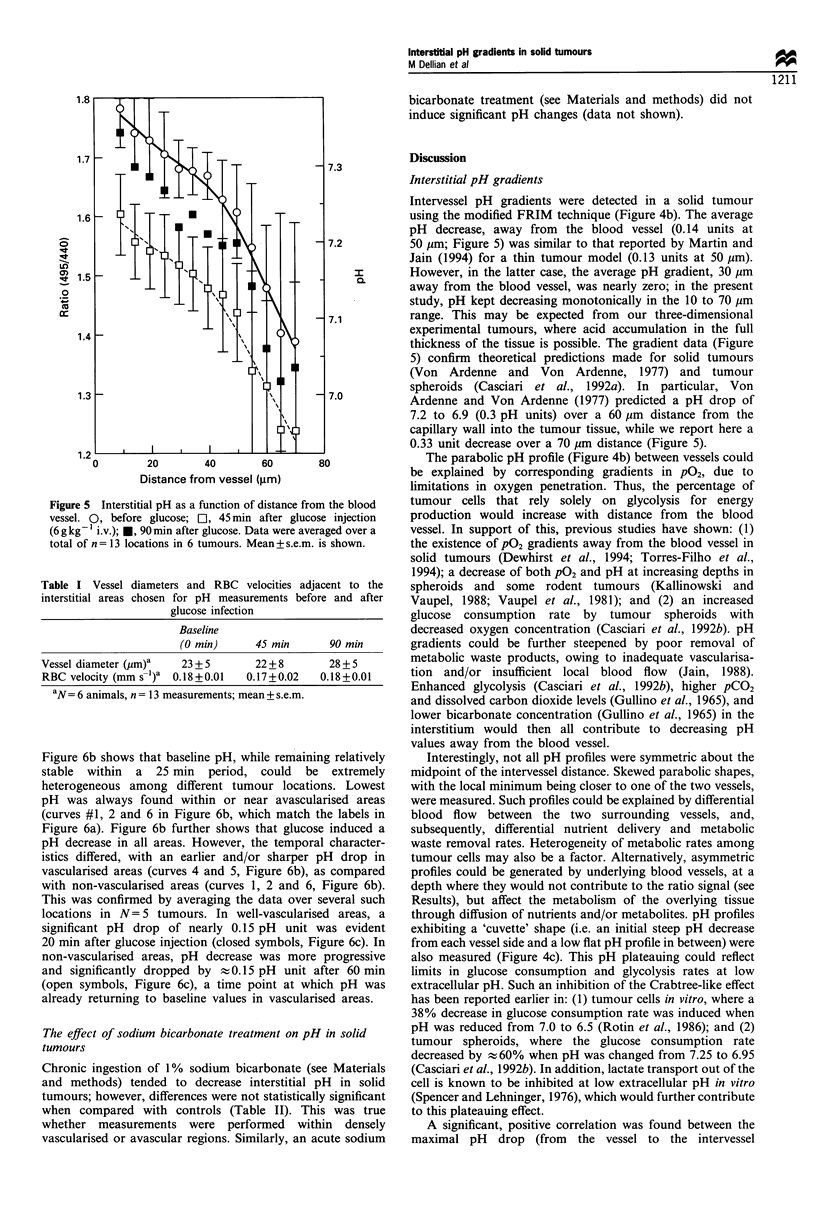

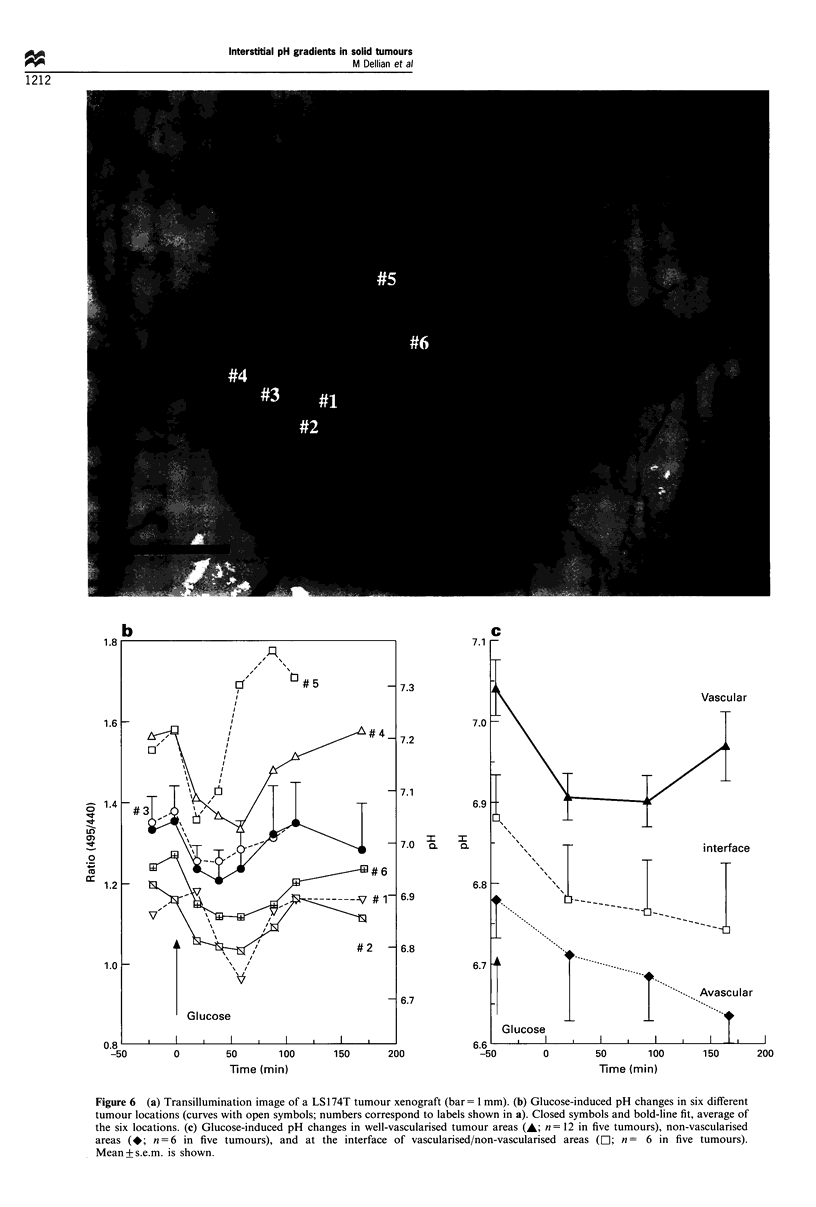

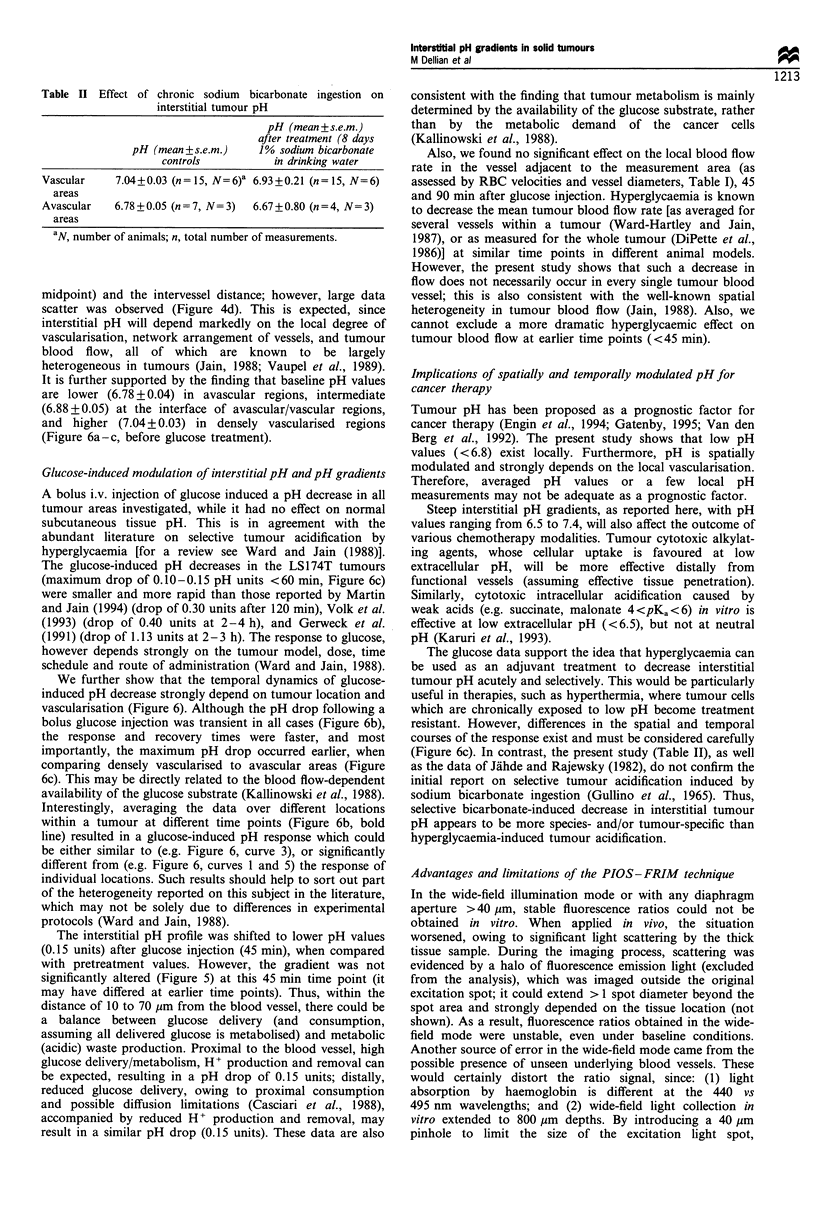

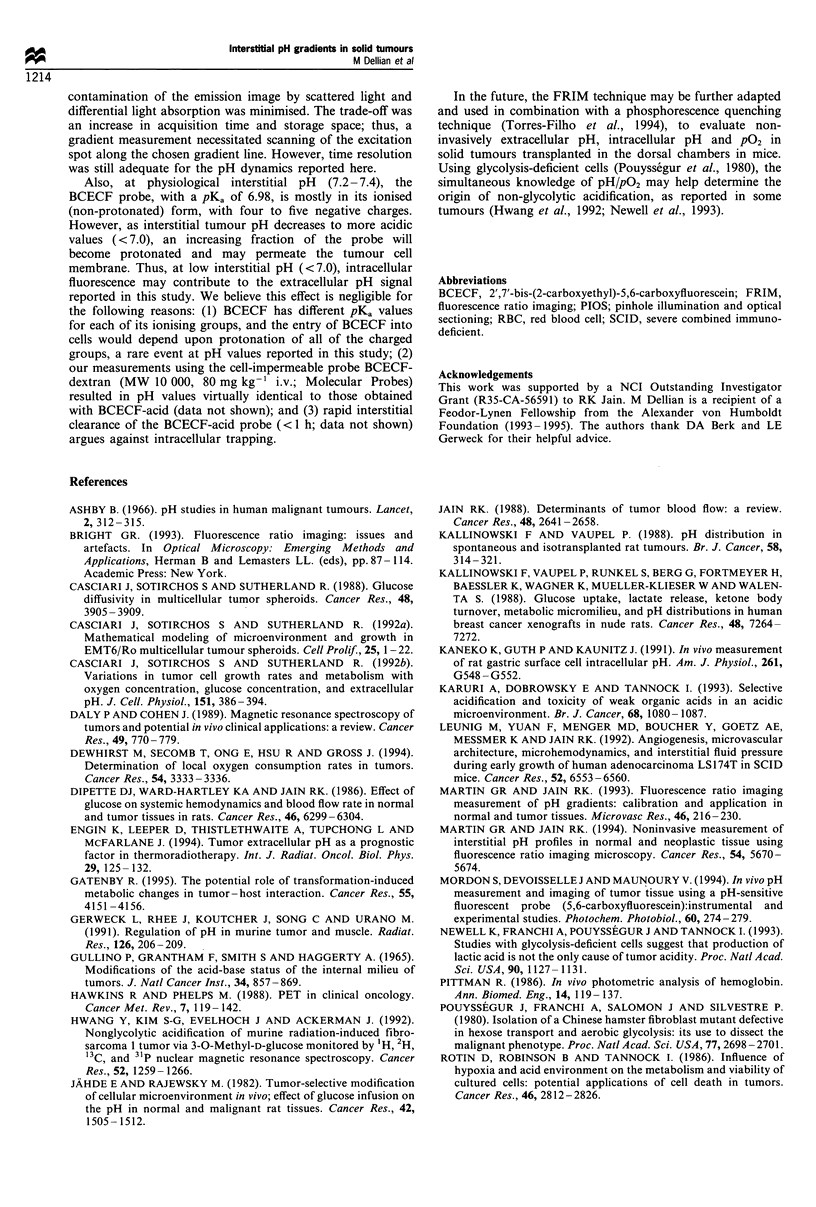

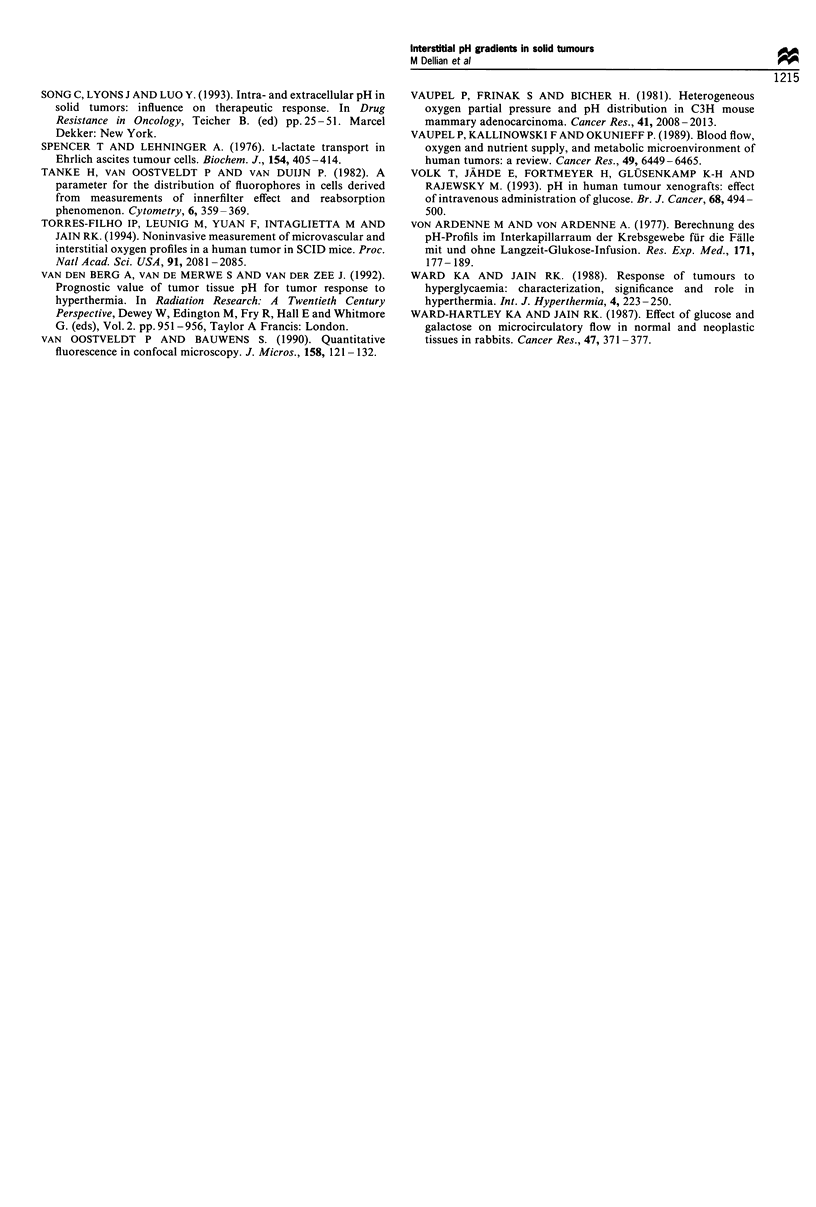


## References

[OCR_01198] Ashby B. S. (1966). pH studies in human malignant tumours.. Lancet.

[OCR_01208] Casciari J. J., Sotirchos S. V., Sutherland R. M. (1988). Glucose diffusivity in multicellular tumor spheroids.. Cancer Res.

[OCR_01213] Casciari J. J., Sotirchos S. V., Sutherland R. M. (1992). Mathematical modelling of microenvironment and growth in EMT6/Ro multicellular tumour spheroids.. Cell Prolif.

[OCR_01215] Casciari J. J., Sotirchos S. V., Sutherland R. M. (1992). Variations in tumor cell growth rates and metabolism with oxygen concentration, glucose concentration, and extracellular pH.. J Cell Physiol.

[OCR_01221] Daly P. F., Cohen J. S. (1989). Magnetic resonance spectroscopy of tumors and potential in vivo clinical applications: a review.. Cancer Res.

[OCR_01226] Dewhirst M. W., Secomb T. W., Ong E. T., Hsu R., Gross J. F. (1994). Determination of local oxygen consumption rates in tumors.. Cancer Res.

[OCR_01231] DiPette D. J., Ward-Hartley K. A., Jain R. K. (1986). Effect of glucose on systemic hemodynamics and blood flow rate in normal and tumor tissues in rats.. Cancer Res.

[OCR_01236] Engin K., Leeper D. B., Thistlethwaite A. J., Tupchong L., McFarlane J. D. (1994). Tumor extracellular pH as a prognostic factor in thermoradiotherapy.. Int J Radiat Oncol Biol Phys.

[OCR_01244] Gatenby R. A. (1995). The potential role of transformation-induced metabolic changes in tumor-host interaction.. Cancer Res.

[OCR_01247] Gerweck L. E., Rhee J. G., Koutcher J. A., Song C. W., Urano M. (1991). Regulation of pH in murine tumor and muscle.. Radiat Res.

[OCR_01252] Gullino P. M., Grantham F. H., Smith S. H., Haggerty A. C. (1965). Modifications of the acid-base status of the internal milieu of tumors.. J Natl Cancer Inst.

[OCR_01259] Hawkins R. A., Phelps M. E. (1988). PET in clinical oncology.. Cancer Metastasis Rev.

[OCR_01263] Hwang Y. Y., Kim S. G., Evelhoch J. L., Ackerman J. J. (1992). Nonglycolytic acidification of murine radiation-induced fibrosarcoma 1 tumor via 3-O-methyl-D-glucose monitored by 1H, 2H, 13C, and 31P nuclear magnetic resonance spectroscopy.. Cancer Res.

[OCR_01276] Jain R. K. (1988). Determinants of tumor blood flow: a review.. Cancer Res.

[OCR_01270] Jähde E., Rajewsky M. F. (1982). Tumor-selective modification of cellular microenvironment in vivo: effect of glucose infusion on the pH in normal and malignant rat tissues.. Cancer Res.

[OCR_01285] Kallinowski F., Vaupel P., Runkel S., Berg G., Fortmeyer H. P., Baessler K. H., Wagner K., Mueller-Klieser W., Walenta S. (1988). Glucose uptake, lactate release, ketone body turnover, metabolic micromilieu, and pH distributions in human breast cancer xenografts in nude rats.. Cancer Res.

[OCR_01280] Kallinowski F., Vaupel P. (1988). pH distributions in spontaneous and isotransplanted rat tumours.. Br J Cancer.

[OCR_01294] Kaneko K., Guth P. H., Kaunitz J. D. (1991). In vivo measurement of rat gastric surface cell intracellular pH.. Am J Physiol.

[OCR_01298] Karuri A. R., Dobrowsky E., Tannock I. F. (1993). Selective cellular acidification and toxicity of weak organic acids in an acidic microenvironment.. Br J Cancer.

[OCR_01303] Leunig M., Yuan F., Menger M. D., Boucher Y., Goetz A. E., Messmer K., Jain R. K. (1992). Angiogenesis, microvascular architecture, microhemodynamics, and interstitial fluid pressure during early growth of human adenocarcinoma LS174T in SCID mice.. Cancer Res.

[OCR_01310] Martin G. R., Jain R. K. (1993). Fluorescence ratio imaging measurement of pH gradients: calibration and application in normal and tumor tissues.. Microvasc Res.

[OCR_01315] Martin G. R., Jain R. K. (1994). Noninvasive measurement of interstitial pH profiles in normal and neoplastic tissue using fluorescence ratio imaging microscopy.. Cancer Res.

[OCR_00036] Martin G. R., Jain R. K. (1994). Noninvasive measurement of interstitial pH profiles in normal and neoplastic tissue using fluorescence ratio imaging microscopy.. Cancer Res.

[OCR_01319] Mordon S., Devoisselle J. M., Maunoury V. (1994). In vivo pH measurement and imaging of tumor tissue using a pH-sensitive fluorescent probe (5,6-carboxyfluorescein): instrumental and experimental studies.. Photochem Photobiol.

[OCR_01327] Newell K., Franchi A., Pouysségur J., Tannock I. (1993). Studies with glycolysis-deficient cells suggest that production of lactic acid is not the only cause of tumor acidity.. Proc Natl Acad Sci U S A.

[OCR_01337] Pouysségur J., Franchi A., Salomon J. C., Silvestre P. (1980). Isolation of a Chinese hamster fibroblast mutant defective in hexose transport and aerobic glycolysis: its use to dissect the malignant phenotype.. Proc Natl Acad Sci U S A.

[OCR_01343] Rotin D., Robinson B., Tannock I. F. (1986). Influence of hypoxia and an acidic environment on the metabolism and viability of cultured cells: potential implications for cell death in tumors.. Cancer Res.

[OCR_01358] Spencer T. L., Lehninger A. L. (1976). L-lactate transport in Ehrlich ascites-tumour cells.. Biochem J.

[OCR_01364] Tanke H. J., van Oostveldt P., van Duijn P. (1982). A parameter for the distribution of fluorophores in cells derived from measurements of inner filter effect and reabsorption phenomenon.. Cytometry.

[OCR_01371] Torres Filho I. P., Leunig M., Yuan F., Intaglietta M., Jain R. K. (1994). Noninvasive measurement of microvascular and interstitial oxygen profiles in a human tumor in SCID mice.. Proc Natl Acad Sci U S A.

[OCR_01385] Vaupel P. W., Frinak S., Bicher H. I. (1981). Heterogeneous oxygen partial pressure and pH distribution in C3H mouse mammary adenocarcinoma.. Cancer Res.

[OCR_01392] Vaupel P., Kallinowski F., Okunieff P. (1989). Blood flow, oxygen and nutrient supply, and metabolic microenvironment of human tumors: a review.. Cancer Res.

[OCR_01398] Volk T., Jähde E., Fortmeyer H. P., Glüsenkamp K. H., Rajewsky M. F. (1993). pH in human tumour xenografts: effect of intravenous administration of glucose.. Br J Cancer.

[OCR_01412] Ward-Hartley K. A., Jain R. K. (1987). Effect of glucose and galactose on microcirculatory flow in normal and neoplastic tissues in rabbits.. Cancer Res.

[OCR_01407] Ward K. A., Jain R. K. (1988). Response of tumours to hyperglycaemia: characterization, significance and role in hyperthermia.. Int J Hyperthermia.

[OCR_01401] von Ardenne M., von Ardenne A. (1977). Berechnung des pH-Profils im Interkapillarraum der Krebsgewebe für die F¿lle mit und ohne Langzeit-Glukose-Infusion.. Res Exp Med (Berl).

